# Relative adrenal insufficiency in adults with sickle cell disease

**DOI:** 10.11604/pamj.2018.29.30.6025

**Published:** 2018-01-12

**Authors:** Eugene Sobngwi, Noel Désirée Mbango, Eric Vounsia Balti, Francoise Ngo Sack, Vicky Ama Moor, Jean-Claude Mbanya

**Affiliations:** 1 Department of Internal Medicine, Endocrine and Diabetes Unit, Yaoundé Central Hospital, Yaoundé, Cameroon; 2 Faculty of Medicine Biomedical Sciences, University of Yaoundé 1, Yaoundé, Cameroon; 3 Faculty of Medicine and Pharmacy, Brussels Free University-VUB, Brussels, Belgium; 4 Hematology and Oncology Unit, Yaoundé Central Hospital, Yaoundé, Cameroon

**Keywords:** Adrenal function, sickle cell disease, adult, cortisol, tetracosactide

## Abstract

Rheological modifications observed in sickle cell anemia are associated with ischemic complications that can cause target organ functional impairment. The objective was to investigate adrenal function of adult patients with sickle cell disease. In this cross-sectional study conducted in a tertiary referral hospital of the capital city of Cameroon, we enrolled ten crisis-free adult patients with sickle cell disease (SCD) and ten age- and sex-matched healthy individuals. We assessed adrenal function by testing basal cortisol levels and 60 min after tetracosactide (Synacthen^®^) injection using immuno-chemiluminescence method. Post-stimulatory cortisol was defined as primary endpoint and secondary endpoints include basal cortisol levels, post-stimulatory cortisol increments and the fold increase of cortisol one hour after stimulation. Sickle cell patients had an impairment of adrenal function despite no significant difference between patients’ and controls’ for basal or post-stimulatory cortisol levels. In fact, one patient in two failed to achieve a two-fold increase in cortisol levels after stimulation (5/10) as opposed to 1 in 10 in the control population (1/10), *P* = 0.070. The percent increment of cortisol after stimulation was lower in patients versus controls (133 vs 207, *P* = 0.047). Relative adrenal insufficiency is frequent in sub-Saharan adult patients with sickle cell disease despite normal basal cortisol levels. Our results suggest that adrenal function require further investigation during SCD crises as these represent an important stress and may worsen the prognosis.

## Introduction

Sickle cell disease (SCD) is an autosomal recessive disease that results from a point mutation on the beta-globin molecule leading to erythrocyte rigidity and increased episodes of vaso-occlusive crisis [1]. Under precipitating conditions, red blood cell sequestration and hemolysis give rise to the clinical presentation of the disease mainly marked by anemia, pain and multi-organ ischemic damage [2]. Recurrence of vaso-occlusive episodes has been associated to cerebro-vascular ischemic events, renal damage, pulmonary ischemia and alteration of some endocrine functions in SCD [3,4]. Beside red blood cell sequestration and the resulting ischemia, the pathophysiology entails iron overload which has been associated to oxidative stress and multi-organ failure [5,6]. Previous studies have addressed the question of endocrine function in SCD [4]. Nevertheless, there is currently to the best of our knowledge, insufficient data on adrenal function in sickle cell patients, specifically in sub-Saharan Africa, the most affected part of the world with the highest SCD-related mortality. We therefore aimed in this study at investigating adrenal function of adult individuals with SCD in Cameroon.

## Methods

### Study population

Crisis-free adult (> 18 years old) patients with SCD were enrolled over a period of seven months (June-December 2007). The study was conducted in Yaoundé Central Hospital, one of the four tertiary reference hospitals in the capital city of Cameroon. Corticosteroid administration in the last three months preceding the investigation, recent infection, coagulopathy, thrombocytopenia, ongoing treatment including medications that could impact on adrenal function (anti-TB drugs, anti-epileptic therapy…) and recent episode of vaso-occlusive crisis were exclusion criteria. Age- and sex-matched healthy volunteers with normal hemoglobin genotype (HbAA) were recruited through advertisement among the general population as control individuals. Written informed consent was obtained from all participants. The study was approved by the institutional review board and the National Ethics Committee. Anthropometric and clinical data were collected via a standard questionnaire and clinical examination.

### Assessment of adrenal function

Adrenal function was assessed after an overnight 12-hour fast. Blood sample was drawn from the antecubital vein prior to an IM injection of 0.25μg of tetracosactide (Synacthen^®^ 0.25mg/ml, Sigma-tau, Rome, Italy) and 60 min after injection for assessment of plasma cortisol levels [7]. Cortisol levels were measured in duplicate by immuno-chemiluminescence (Immulite^®^ 2000 Immunoassay System, Siemens, Los Angeles, CA, USA). The detection sensitivity of the method is 5.5 nmol/l and intra- / inter-assay coefficient of variation obtained from two series of samples analyzed daily in duplicate during 20 days were < 5%

### Statistical analysis

Statistical differences between groups were determined by independent samples *t*-test or Wilcoxon’s rank test for continuous data and Fischer’s exact test in the case of categorical variables. The minimum required sample size (n = 8) was calculated to detect a 20% difference from a mean plasma cortisol level of 430.40 nmol/l. The chosen cut-off value for type I was 5% to reach an 80% power using 95% confidence interval (CI). The primary endpoint in this study was plasma cortisol level 60 min after stimulation and secondary endpoints include basal cortisol level, post-stimulatory incremental cortisol variation and cortisol fold increase. The post-stimulatory incremental cortisol variation (?cortisol) was estimated as the proportion of the difference between cortisol levels 60 min after stimulation and basal cortisol values. Statistical tests were performed two-tailed using SPSS for windows version 17.0 (SPSS, Chicago, IL, USA). Results with *P* value < 0.05 were considered significant.

## Results

Twenty crisis-free SCD patients and healthy volunteers were included in the study with a 1:1 ratio and a 60% proportion of male in each group. Systolic and diastolic blood pressure were similar in patients and controls while patients’ body fat composition tended to be lower in the presence of SCD (*P* = 0.080). Patients had on average 3 vaso-occlusive crises within the last twelve. SCD patients were significantly leaner than control individuals (*P* = 0.030). None of them had received a blood transfusion in the last 12 months. The characteristics of the study population are shown in Table 1. Although baseline cortisol levels were normal in all patients and comparable to healthy volunteers (286.9±79.1 vs. 263.6±64.7 nmol/L, *P* = 0.900). Patients’ mean post-stimulatory cortisol value was lower than control participants although the difference was not significant (*P* = 0.090). However, the incremental cortisol variation after tetracosactide stimulation was significantly lower in the presence of SCD (132.8 vs. 207.36, *P* = 0.047). Overall, five out of ten (50%) patients failed to achieve an optimal adrenal stimulation by reaching a two fold increase of basal plasma cortisol levels.

**Table 1:** General characteristics and adrenal function of the study population

Parameters	Sickle cell patients	Healthy controls	*P*-value
	(n=10)	(n=10)	
Age, years	28± 5	28± 5	1.000
Gender, M/F (ratio)	6/4 (1.5)	6/4 (1.5)	1.000
BMI, kg/m^2^	19.4±1.3	23.7±2.0	0.030
SBP, mmHg	105±11	113±16	0.200
DBP, mmHg	58±12	70±13	0.400
Body fat composition, %	10.6±8.1	18.1±6.0	0.080
Clinical findings			
Vaso-occlusive crisis <12 months,	3 (1-5)	na^a^	na
All vaso-occlusive crisis^b^,	40 (20-75)	na	na
Heart murmures, n (%)	6 (60)	0 (0)	0.011
Hepatomegaly, n (%)	3 (30)	0 (0)	0.211
Splenomegaly, n (%)	3 (30)	0 (0)	0.211
Jaundice, n (%)	6 (60)	0 (0)	0.011
Biological data			
Basal cortisol, nmol/l	286.9±79.1	263.6±64.7	0.900
Post-stimulation cortisol, nmol/l	674.3±149.3	810.1±174.9	0.090
Two-fold increase after stimulation, n (%)	5 (50)	9 (90)	0.070
∆cortisol ^c^, %	132.8	207.4	0.047

Data are expressed as mean ± standard deviation/median (inter-quartile range) or frequencies as count (proportion);

a not applicable;

b total number of vaso-occlusive crisis since diagnosis;

c incremental cortisol variation after low dose ACTH stimulation; BMI: body mass index; SBP: systolic blood pressure; DBP: diastolic blood pressure.

## Discussion

The findings suggest that relative adrenal insufficiency is not an infrequent event in crisis-free sickle cell patients. Although both basal and post-stimulatory cortisol levels were normal, the incremental cortisol variation was significantly lower in patients. Moreover, in the presence of SCD, the vast majority of participants failed to achieve significant increase in basal cortisol level after stimulation.

Based on our results patients with SCD are leaner than healthy controls. Because none of the patients had a recent intercurrent disease, the difference in BMI may solely be due to underlying metabolic changes due to the disease. In line with this, Borel et al. reported a 15% accretion of resting energy expenditure (REE) as well as an excess of protein breakdown in African-American patients with SCD compared to control in individuals [8]. Interestingly, the same excess of energy expenditure was observed in the presence of exogenous energy supply [9]. Moreover, even in the absence of crises, REE has been correlated with low-grade inflammation and oxidative stress in SCD [10]. This suggests that a higher energy and protein intake may not only correct the leaner body feature but could also reduce debilitating effects of iron-overload in sickle cell patients.

Furthermore, it is apparent that patients failed more often to double their cortisol levels after stimulation and the median increase in cortisol values was lower than in individual carrying normal hemoglobin genotype. Basal plasma cortisol level has previously been reported to be lower in SCD children compared to both individuals with HbAA and HbAS genotypes [11]. In fact, endocrine dysfunctions are known to be the most frequent event occurring in sickle cell anemia and the impairment occurs as a result of recurrent occlusion of small vessels but also as an outcome of oxidative stress mediated organ toxicity led by iron-overload [12]. Therefore, one would expect the level of organ damage - in the case of our study adrenal insufficiency - to be correlated with the frequency of blood transfusions. Although none of the patients described in our report received blood during the past 12 months, it is worth mentioned that included individuals were above age 18; thus the observed relative adrenal insufficiency could occur as an outcome of chronic insidious hemolytic events and oxidative stress due to gradual iron accumulation [5,6].

The limitations of our study include the small sample size; although appropriate and well powered, a bigger study population may have enhanced the magnitude of estimates. Also, we do not report on basal plasma ACTH levels that could reveal the primary or secondary origin of the adrenal function impairment; 3. The proportion of SS hemoglobin in patients has not been assessed; this may have given some clues on the association with the frequency of vaso-occlusion events thus aid in understanding the relationship with the resulting adrenal insufficiency [1]. Finally we lack data on markers of oxidative stress and iron-overload to show the association with the low BMI observed in our study population. This is however a broader scope than what was foreseen by this study. Despite these limitations, we performed a stimulatory test which is a strong asset worth noticed.

## Conclusion

Our findings suggest that relative adrenal insufficiency is not a rare event in crisis-free adult patients with SCD. We believe that this study may pave the way to further investigations of adrenal function during vaso-occlusive crisis and to subsequent studies aiming at unraveling the interplay between iron-overload, acute low-grade inflammation, oxidative stress and adrenal insufficiency in SCD patients in the presence or not of vaso-occlusive events.

### What is known about this topic

- The propensity to thromboembolic events and iron deposition could potentially affect adrenal function in patients with SCD;
- There is potentially an exacerbation of adrenal dysfunction in patients with SCD with subcritical hypoadrenalism during sickle cell crisis which could unravel a clinically overt adrenal insufficiency;
- There is a paucity of data comparing cortisol response after stimulation in crisis-free patients with SCD and healthy (non-SCD) individuals.

### What this study adds

- This study sought to compare stimulated cortisol response of crisis-free patients with SCD to age- and sex-matched non-SCD controls;
- We find a relative difference in adrenal response in crisis-free patients with SCD compared to the control population but not to a level suggesting a higher tendency to adrenal insufficiency in patients with SCD;
- These data suggest that further investigation in a larger population and comparisons of these findings with those in SCD patients during vaso-occlusive crisis is needed for a better understanding of the potential disease impact on pituitary-adrenal function.

## Competing interests

The authors declare no competing interests.

## Authors’ contributions

All authors have read and agreed to the final manuscript.
